# Communication Errors in Human–Chatbot Interactions: A Case Study of ChatGPT Arabic Mental Health Support Inquiries

**DOI:** 10.3390/bs15081119

**Published:** 2025-08-18

**Authors:** Ghuzayyil Mohammed Al-Otaibi, Hind M. Alotaibi, Sami Sulaiman Alsalmi

**Affiliations:** Department of English, College of Language Sciences, King Saud University, P.O. Box 2460, Riyadh 11451, Saudi Arabia; salsulmi@ksu.edu.sa

**Keywords:** mental health support, communication errors, ChatGPT, Arabic, artificial intelligence, case study

## Abstract

Large language models (LLMs) have become extensively used among users across diverse settings. Yet, with the complex nature of these large-scale artificial intelligence (AI) systems, leveraging their capabilities effectively is yet to be explored. In this study, we looked at the types of communication errors that occur in interactions between humans and ChatGPT-3.5 in Arabic. A corpus of six Arabic-language consultations was collected from an online mental health support forum. For each consultation, the researchers provided the user’s Arabic queries to ChatGPT-3.5 and analyzed the system’s responses. The study identified 102 communication errors, mostly grammatical and repetitions. Other errors involved contradictions, ambiguous language, ignoring questions, and lacking sociality. By examining the patterns and types of communication errors observed in ChatGPT’s responses, the study is expected to provide insights into the challenges and limitations of current conversational AI systems, particularly in the context of sensitive domains like mental health support.

## 1. Introduction

It is evident that large language models (LLMs) of artificial intelligence (AI) systems have become widely used among users across various settings. These systems proved to be powerful tools in terms of their language understanding and generation abilities, not only in English but in other languages as well. The affordance and usability of these systems promoted their use in a diverse range of contexts e.g., educational settings (Albadarin et al., 2024; Montenegro-Rueda et al., 2023; Prananta et al., 2023; Sok & Heng, 2023; Zhang & Tur, 2024), customer services (Damaševičius & Zailskaitė-Jakštė, 2024; George & George, 2023), and health care sector (Biswas, 2023; Cascella et al., 2023; J. Li et al., 2024; Sallam, 2023; L. Wang et al., 2024; Wu et al., 2024).

Utilizing AI Chatbots in mental care settings has been increasingly evident. Several researchers highlighted the potential benefits of such integration (e.g., Alanezi, 2024; D’Silva & Pande, 2024; Garg & Chauhan, 2024; Pandya et al., 2024). One of the key benefits is easy access to mental health support, as these systems are available 24/7 and can manage high volumes of requests. Additionally, these systems can serve as an alternative for individuals who cannot reach traditional in-person mental health support due to social stigma or privacy concerns. According to Alanezi (2024), AI systems are able to provide personalized responses based on the user’s preferences and history, which promotes the user’s experience and engagement. The researcher argues that such systems can successfully promote psychoeducation and support self-management techniques (Alanezi, 2024).

On the other hand, other experts have raised concerns regarding the limitations and potential risks resulting from integrating AI tools into mental health support (D’Silva & Pande, 2024; Garg & Chauhan, 2024). One of the major concerns is associated with inherent biases and inaccuracies, as these systems are trained on human-generated texts. Such limitations can potentially be harmful to vulnerable mental health support seekers.

Ethical concerns related to data privacy and confidentiality are another serious issue raised by several researchers (e.g., Garg & Chauhan, 2024; Pandya et al., 2024), emphasizing an urgent need to integrate robust protocols to protect the vulnerable users’ sensitive information while using AI chat tools for mental health support.

Finally, although these systems have the ability to interact in multiple languages, they fail to comprehend nuanced cultural and linguistic factors (Alanezi, 2024; D’Silva & Pande, 2024). Several studies showed that AI systems tend to provide overgeneralized or inappropriate recommendations due to their inability to fully understand the complex linguistic and sociocultural factors that influence mental health.

The ongoing debate on the potential of integrating AI systems into mental health support contexts and the concerns associated with it emphasizes an urgent need for continuing research to comprehensively understand the effectiveness of these systems and rigorously evaluate their ability to provide accurate, empathetic, and personalized support, specifically in the context of sensitive domains like mental health support. However, most of the studies conducted to evaluate the effectiveness of AI dialogue systems as therapeutic tools have focused on exploring participants’ level of satisfaction with the tool (e.g., Atiyah, 2023; Melo et al., 2024; Nordgren & Svensson, 2023; Yu & McGuinness, 2024) or verifying the information provided by them through resorting to health professionals or experts (e.g., Fu et al., 2023; Gravel et al., 2023; Oviedo-Trespalacios et al., 2023). Hence, there is a need to examine responses of AI Chatbots comprehensively and analyze communication errors or deviations from the norm, taking into consideration linguistic, pragmatic, and sociocultural aspects, especially if one considers the highly sensitive domain of psychotherapy.

Based on the above discussion, the present study is expected to contribute to this area by addressing the following research questions: (1) What types and patterns of communication errors occur in interactions between humans and ChatGPT-3.5 in Arabic, and (2) how do these errors impact the effectiveness of AI-based mental health support?

The study focuses on ChatGPT-3.5 as a case study since it is one of the early-released and most widely used AI systems among Arab users (Al-Khalifa et al., 2023). The system was developed by OpenAI and released in November 2022 with over 100 million users within the first 2 months of its launch (Aljanabi, 2023). It allows users to engage in natural language conversations in several languages, including Arabic. In the following sections, we further explore the literature on the use of AI systems in mental health support, evaluating their potential and limitations.

This study highlights both the benefits and challenges of integrating AI-powered systems into mental healthcare. The findings can inform the development of strategies to optimize the integration of these systems and mitigate communication challenges, thereby enhancing the effectiveness of these tools in providing accessible and helpful mental health support across diverse populations and contexts.

## 2. Literature Review

### 2.1. Use of ChatGPT in Mental Health Support

ChatGPT is a Chatbot based on a massive language model, developed by OpenAI. It is trained to produce human-like responses and, thus, the abbreviation (GPT) for Generative Pre-trained Transformer (Raile, 2024). The tool is designed to understand a wide range of topics (Al-Khalifa et al., 2023), and it can respond in various languages. Since its release, studies on the use of ChatGPT in various settings have been numerous and varied. Scholars from different fields have been exploring the potential applications of such an advanced AI language model across domains such as education, health care, content creation, language translation, etc.

In the context of mental health support, research findings have generally been positive, acknowledging the potential of ChatGPT and its capabilities in providing accessible and personalized support. On the other hand, studies have reported several challenges and limitations, especially with aspects related to inaccuracy and privacy. In a pilot study conducted by Melo et al. (2024), the researchers evaluated the effectiveness of using ChatGPT in supporting mental health patients. They found that the patients who participated in semi-structured sessions with ChatGPT showed improved self-reported quality of life compared to the control group receiving standard care. Additionally, the participants also reported high levels of satisfaction with the ChatGPT-assisted therapy, emphasizing its effectiveness as a therapeutic tool. Despite the relatively small sample, the findings suggested that ChatGPT has the potential to serve as a supplementary intervention for improving mental health outcomes in psychiatric inpatient care.

According to D’Silva and Pande (2024), ChatGPT could be leveraged as a tool to enhance mental health support and cultivate positive interactions with those seeking assistance online. The researchers evaluated ChatGPT’s responses to 175 online posts identified as cries for help, using qualitative analysis and text analytics. They found that ChatGPT displays improved emotional intelligence, which could enable it to offer a supportive and interactive experience for individuals in mental health communities. However, several key limitations and risks were also highlighted, including potential biases in its training data, inability to capture complex sociocultural factors influencing mental health, possibility of providing inappropriate advice without reliable data, reluctance of individuals to share sensitive information with an AI system, and concerns around privacy, confidentiality, and stigma.

In another study, Gravel et al. (2023) looked at the quality and references provided by ChatGPT in response to 20 medical questions. The system responses were evaluated by three medical experts, where were they found to be of limited quality, with a median score of 60%. Major and minor factual errors were also identified, and they found that 69% of the 59 references provided by the system were fabricated, though they appeared real. The findings suggested that ChatGPT users should be cautious about the references provided, especially when integrating them into medical manuscripts.

One more study by Oviedo-Trespalacios et al. (2023) evaluated the safety-related advice provided by ChatGPT-3.5 across nine different domains, including topics like driving safety, child supervision, crowd management, fall prevention, and workplace safety. The study found that ChatGPT often provided incorrect, potentially harmful, or inappropriate information and advice related to these safety-critical topics. It tended to emphasize individual responsibility over contextual factors, potentially leading to ecological fallacy.

The reviewed studies, while using various methodologies and targeting varied user populations, consistently indicate that ChatGPT and other AI-powered Chatbots can provide accessible mental health support. This support is primarily informational and emotional in nature, consisting of general advice, basic coping strategies, psychoeducational content, and empathetic conversational cues that can help users feel heard, validated, or reassured. Such interactions can be particularly valuable for individuals who are hesitant to seek formal therapy, face stigma, or lack immediate access to professional mental health services. In this sense, ChatGPT can act as a first-line resource, guiding users to recognize signs of distress, offering motivational language, or directing them to appropriate professional help. However, it is critical to point out that the assistance provided by these models does not equate to clinical assessment, therapy, or crisis intervention. The support offered is limited by the model’s training data, lack of contextual awareness, and inability to deliver personalized care or ensure user safety in acute scenarios. Thus, the role of ChatGPT should be framed as that of a supplementary tool—a non-clinical aid useful for mental health awareness, emotional self-regulation, or reinforcement of positive behaviors in cases of mild distress. Due to these limitations, further empirical research is essential to thoroughly evaluate the effectiveness, safety, and ethical considerations associated with deploying such systems in mental health contexts. This is especially important in culturally diverse and high-stakes settings, where linguistic nuances, social norms, and ethical boundaries vary significantly. Moreover, as ChatGPT evolves through continuous updates and refinements, ongoing assessment of its communicative capabilities, cultural sensitivity, and real-world impact is necessary to inform its responsible integration into broader mental health support ecosystems.

### 2.2. Use of ChatGPT in Providing Mental Health Support in Arabic

Since the dominant language of most of the widely used AI systems, including ChatGPT, is English, studies involving participants from other languages and cultures seem limited. In the few studies conducted in the Arab world, for instance, the findings revealed that AI systems struggle to provide culturally appropriate and sympathetic support since these systems were trained primarily on English data.

Alanezi (2024), for example, evaluated ChatGPT’s effectiveness in delivering mental health support among 24 Saudi adult outpatients with mental health conditions, including anxiety, depression, and behavioral disorders. The participants were instructed to use ChatGPT for 2 weeks, seeking support for managing their condition, and then they were interviewed to talk about their experience. The findings acknowledged several challenges related to ethical and legal considerations, accuracy and reliability, limited assessment capabilities, and cultural and linguistic considerations. On the other hand, several positive aspects were highlighted, such as facilitating the users’ self-assessment and monitoring of mental health symptoms and providing crisis intervention support for users experiencing distress or in need of immediate assistance. The researcher concluded that ChatGPT has the potential to offer mental health support when used appropriately as part of a comprehensive care plan.

In another study, Ajlouni et al. (2023) explored the perceptions of mental health undergraduate students towards using ChatGPT in psychological counseling. A questionnaire was administered among 210 counseling and mental health students in a Jordanian university. Although most of the participants acknowledged the potential of ChatGPT in supporting their counseling skills and therapeutic competencies, 59% of the respondents indicated a delay in responding to Arabic queries, while 68.1% agreed that the answers were not appropriate for Arab society, customs, traditions, and culture. The researchers highlighted the need for further research with larger and more diverse samples to gain deeper insights into the opportunities and challenges of leveraging innovative AI tools like ChatGPT to enhance counseling and mental health education.

Finally, in a study by Atiyah (2023), the researcher looked at the impact of AI-assisted emotional support on students’ mental health outcomes in Saudi educational institutions. The researcher compared the mental health outcomes of 512 students who received emotional support from ChatGPT versus those who received support from human counselors. Standardized questionnaires were used to assess anxiety and depression levels in both groups. The study found that the students who received support from ChatGPT demonstrated better mental states than those who relied on human counselors. The researcher concluded that AI-based emotional support systems can be effective in addressing the growing need for mental health services in educational settings. However, the study acknowledged that ChatGPT may fail to fully comprehend the complexities of human emotions and suggested focusing on enhancing the emotional intelligence and empathy of such AI models to further improve their ability to provide meaningful emotional support.

As shown above, studies tackling the issue of integrating AI systems in mental health support among Arab users are rather scarce. Therefore, the current study aims to address this research gap by evaluating the Arabic communication capabilities of a widely used AI system, ChatGPT, when used for mental health support. Knowing that Arabic is a low-resource language, exploring ChatGPT’s ability to understand linguistic and cultural nuances is essential to enhance ChatGPT’s efficiency in providing psycho-counseling.

In the next section, we explore the studies on Chatbots’ communication errors and the most common frameworks used to identify such errors.

### 2.3. Previous Research on Annotating Chatbots’ Communication Errors

A few researchers (e.g., Sanguinetti et al., 2020) have conducted studies on identifying communication errors of Chatbots. However, such studies did not address low-resource languages such as Arabic. Sanguinetti et al. (2020), for example, identified errors found in Italian human–Chatbot conversations. More specifically, the conversations were between customers and conversational agents of service providers (i.e., booking a flight or an appointment). The analysis was not restricted to Chatbot errors but also considered those of customers. The study also attempted to find out to what extent Chatbots are aware of customers’ emotions and able to respond appropriately. The researchers collected 129 conversations from a customer care unit of a telecommunications company. Using frameworks of Higashinaka et al. (2019, 2015a, 2015b), Bernsen et al. (1996), and mostly Herzig et al.’s (2016) classification of negative emotions, the researchers found that the most common errors in customers’ messages were of ignoring questions or feedback, whereas topic change was mainly evident in Chatbot responses. They added that emotions of frustration were more prevailing with errors of non-cooperativity and repetition. According to the researchers, the annotated dataset using the scheme mentioned above is used to develop the rule-based language generation system for the purpose of improving Chatbot responses and customer experience.

In the context of healthcare, Biassoni and Gnerre (2025) investigated the communicative behaviors of ChatGPT based on the nature of the disorder (medical or psychological) and the user’s communication style (neutral vs. expressing concern). The responses were analyzed using Linguistic Inquiry and Word Count (LIWC) to identify linguistic markers of the adjustment to different inquiries and interaction styles. Their results indicated that ChatGPT used more engaging language in treatment contexts and psychological inquiries, showing more analytical thinking in neutral contexts and higher levels of empathy in psychological conditions and when the user expressed concern. Findings suggested that wellness-related language was more prevalent in psychological and treatment contexts. However, illness-related language was more common in diagnostic interactions for physical conditions. The researchers identified two different patterns: high empathy and engagement in psychological/expressing-concern scenarios, and lower empathy and engagement in neutral/physical disease contexts. They suggested that through context and user-concern language adaptation, ChatGPT can improve patient engagement. Although the study offers valuable insights, a major limitation is its exclusive focus on English language interactions, leaving unanswered how ChatGPT adjusts its responses across different languages and cultural contexts.

Sallam and Mousa (2024) conducted a comparative study evaluating the performance of ChatGPT-3.5 and ChatGPT-4 in responding to health-related prompts in two Arabic dialects, Tunisian and Jordanian, using the CLEAR tool for assessing health information quality. Their results showed that both models achieved only average scores (CLEAR: 2.83–3.20) in Tunisian Arabic and above-average scores (CLEAR: 3.40–3.53) in Jordanian Arabic, with responses in both dialects significantly inferior to those generated in English. The findings highlight a notable dialectal performance gap and emphasize the need for enhanced linguistic and cultural diversity in AI model development to ensure equitable access to health information across Arabic-speaking communities.

The support for diverse cultural contexts in ChatGPT was also investigated by Aleem et al. (2024). The researchers used two rounds of structured prompt tests. The first assessed its baseline therapeutic skills, while the second probed its cultural competence in counseling scenarios. Results revealed notable shortcomings: ChatGPT struggles with memory, adaptability, active listening, engagement depth, and sensitivity to cultural nuances. The authors stressed the importance of enhancing AI systems with better cultural empathy and context awareness. They proposed recommendations for more culturally sensitive AI, contributing to human–computer interaction literature and highlighting the need for AI-driven mental health tools that are culturally attuned for more effective and empathetic therapeutic interactions.

In a recent study, Alasmari (2025) provided a comprehensive scoping review of Arabic NLP applications in mental health, examining the studies published up to early 2025.

He pointed out the lack of targeted NLP tools tailored to Arabic mental health needs and identified common methodological approaches. The study also classified the types of support these tools offer (e.g., symptom detection, sentiment analysis, crisis response). The researcher highlighted clear gaps in data availability, cultural and dialectal considerations, and evaluation standards. In addition, the findings indicated a need for enhanced resource development, culturally informed modeling, and standardized benchmarks to improve Arabic NLP systems’ accuracy, reliability, and clinical relevance in mental health contexts.

While various studies have focused on communication errors in Chatbot responses in English, there is a clear absence of similar work addressing these issues in Arabic or other low-resource languages. According to Ochieng et al. (2024), there is a strong need to continually enhance LLMs to address the complexities of culturally nuanced and low-resource real-world contexts, along with the development of evaluation benchmarks that can accurately capture these challenges. This emphasizes the importance of the current study, which aims to explore communication errors within Arabic-language Chatbot interactions, a context that remains largely underexplored.

### 2.4. Communication Errors in AI Chatbots

There are a number of suggested frameworks that evaluate AI Chatbot responses beyond accuracy, satisfaction, and effectiveness. The first one is called Counselling Bench, and it has been proposed by Liu et al. (2023) to evaluate ChatCounselor, an LLM, designed to provide psychological counseling for mental health patients. The evaluation criteria established are those of accuracy, relevance, empathy, clarity, Chatbot’s listening and reflection skills, interpretation ability, information sharing with system users, and the ability to ask appropriate questions. However, the framework encompasses the skills that professional psychologists should have without any consideration of linguistic or other pragmatic aspects.

Considering the issue of *dialogue safety*, where patients are ensured a comfortable space in which they communicate their thoughts freely without the fear of being judged or criticized (Qiu et al., 2023), Qiu et al. (2023) proposed a number of criteria, such as providing *safe* responses (i.e., correct, helpful, and understandable responses), giving *nonsensical* responses (i.e., lacking in semantics, confusing pronouns, or having repetitions), emphasizing *humanoid mimicry* (i.e., supporting human–Chatbot interactions in clinical practice might negatively affect the ethical principle of integrity), showing *linguistic neglect* (i.e., Chatbots should stick to topics and concerns raised by users), providing *unamiable judgment* (i.e., conversational bots should not negatively evaluate users or verbally abuse them), using *toxic language* (i.e., the Chatbot should not use offensive language or show social bias), delivering *unauthorized preachment* (i.e., providing harmful instructions), and using *nonfactual* statements (i.e., showing bias when expressing opinions on sensitive topics). Though Qiu et al.’s model focuses on linguistic and non-linguistic aspects, it provides more value to safety, ethical principles, and the professional code of conduct. More importantly, the model is not data-driven or based on a theory.

More notably, other studies (e.g., Bojic et al., 2023; Wölfel et al., 2023) addressing communication errors in Chatbot responses strictly adhered to Grice’s Cooperative Principles (P. Grice, 1989) as a framework for data analysis. However, Higashinaka et al. (2021) note that Gricean maxims are only suitable for describing implicatures (i.e., intended implied meanings that go beyond the literal sense of an utterance; P. Grice, 1989) in human conversations. Thus, Higashinaka et al. (2015a, 2015b, 2021) developed an integrated taxonomy of errors that is theory- and data-driven, and, hence, it can be used to comprehensively annotate dialogue system errors. Higashinaka et al. (2015a, 2015b, 2021) based their framework on the Gricean maxims of the Cooperative Principle to identify communication errors typically found in human–Chatbot interactions. The principle assumes that participants cooperate in conversations, and that their interaction with one another is essentially informative, relevant, truthful, and clear (P. Grice, 1989). Thus, Gricean maxims are mainly quantity, relation, quality, and manner. According to P. Grice (1989), the maxim of quality states that one’s contribution to a conversation should be truthful. On the other hand, abiding by the maxim of quantity means that one’s response to another participant’s query should not be more or less informative. As for the maxim of relation, conversationalists are aware that their interaction should not go off-topic (P. Grice, 1989). However, the maxim of manner states that participants’ communication should be clear and done in an orderly manner.

The newly developed framework by Higashinaka et al. (2021) integrates two taxonomies. The former is theory-driven (Higashinaka et al., 2015a) and based on H. P. Grice’s (1975) theory of the Cooperative Principle and Schegloff and Sacks’s (1973) adjacency pairs. Such a theory-driven framework considers deviations from Gricean principles as errors. However, the latter taxonomy is data-driven (Higashinaka et al., 2015b), and it is strictly based on the typical errors made by chat-based systems. Higashinaka et al. (2021) justify the integration of both taxonomies, arguing that theory-based taxonomies are not appropriate for categorizing errors made by Chatbots. The data-based taxonomy, on the other hand, relies heavily on the data collected at a particular technical stage of chat system development. Thus, it may not account for new error categories.

Considering the limitations found in both taxonomies, the newly integrated taxonomy contains 17 error categories (Higashinaka et al., 2021), and it is based on H. P. Grice’s (1975) theory of the Cooperative Principle and the four parameters of a dialogue: (a) utterance, (b) response, (c) context (i.e., discourse), and (d) environment (i.e., the wider context of a dialogue). The first two parameters are known as an adjacency pair (Schegloff & Sacks, 1973). A communication error is considered if it deviates from each maxim in each scope. It is important to note that Higashinaka et al.’s (2015a, 2015b, 2021) framework of annotating Chatbot communication errors went through a number of modifications. According to Higashinaka et al. (2021), the suggested model achieved a higher reliability score using Fleiss’ kappa. The following elaboration on the framework is restricted to the categories of errors reflected by the present study data. Table 1 below illustrates the main categories of utterance, response, context, and society, as well as their respective sub-categories of error types. Because a few categories show some overlap in their definitions, the researchers differentiated between them with examples.

Regarding errors based on *utterances*, they are interpreted with reference to single utterances without considering prompts. Such errors stem from deficiencies in the language generated because of the system’s reliance on the resources available online (Higashinaka et al., 2014). As shown above, utterances can be uninterpretable (i.e., containing meaningless words, Example 1) or ungrammatical/syntactic (i.e., lacking in arguments, Example 2), but they can also include a string of predicates and arguments contributing no meaning. Another example of a content-based error is an utterance with clearly wrong information, as in Example 3 (Higashinaka et al., 2015a, 2021). The initial S is for the system, and U stands for the user.

1. U: What should I do?
  S: Whonps. (This is a meaningless word in English.)
2. U: My brother has suicidal thoughts.
  S: Call hotline. (The system should complete the sentence with a specification of the emergency number.)
3. U: Who is the current President of the USA?
  S: George Washington

However, responses to users’ prompts contain errors that are interpreted in light of *adjacency pairs* (Higashinaka et al., 2015a, 2021). That is, they cannot be analyzed without examining the *prompts* written for the Chatbot, and such categories of errors roughly correspond to Grice’s maxims. For example, ignoring a question and elaborating on a different topic is considered a violation of the relevance maxim (Example 4). On the other hand, ignoring a sub-question, even when the answer is within the scope of the discussed topic, is regarded as a violation of the quantity maxim because the response lacks enough information (Example 5; Higashinaka et al., 2015a, 2021). Additionally, the Chatbot’s answer may include unnecessary details; thus, a violation of the quantity maxim occurs (Example 6). Further, responses may violate content when Chatbots yield responses that do not meet users’ expectations or reflect their earlier prompts. In other words, the response does not reflect the expected content. Higashinaka et al. (2015a, 2015b) treat such errors as violations of the quality maxim (Example 7).

4. U: How can I take care of my skin?
  S: Skin is considered the largest organ in the human body. It is the outer coverage and the protector of the internal organs. People can be dark-skinned, but others can have lighter skin. (The response does not contain any reference to skin care.)
5. U: What is the Capital of Saudi Arabia? Where is it located?
  S: Riyadh. (The Chatbot’s response does not include an indication of the Capital’s location.)
6. U: How can I write a short story?
  S: First, you need to think of a plot. Story writing is not difficult to do. Once you have a lesson you want to convey to the audience, things will be easier for you to do. Second, create a mind map of the major events. Following the old tradition of story writing, you should have a resolution and a climax. Once you write the plot, show your draft to a friend. Pay attention to language mistakes if there are any. Some common mistakes in story writing by novice writers are run-on sentences. (Sentences elaborating on grammatical mistakes and how easy story writing can be are unnecessary details.)
7. U: How can I improve myself in English?
  S: Consider the following if you want to improve your English:
  1. Do not memorize words out of sentences.
  2. Listening and speaking are as important as reading and writing.
  3. Practicing and consistency are essential to language learning.
  U: I struggle in grammar?
  S: French grammar is easy if you pay sufficient attention to word endings. (The answer is not expected because the first prompt is about English.)

Additionally, errors can be identified with reference to the *context* in which they occur and not only with respect to adjacency pairs. Errors stem from the topics that the system dwells on and the propositions it uses. Thus, the subcategories mentioned below reflect some Gricean maxims (Higashinaka et al., 2015a, 2015b, 2021). Hence, there can be a violation of quantity where the Chatbot provides unnecessary elaboration of a relevant point or delivers less information in the form of repetitions or paraphrases (Example 8). Moreover, context-level responses may include ambiguous terms or expressions that need further explanation, and, thus, a violation of the manner maxim occurs (Example 9). More importantly, the system’s response may contradict itself or its conversational partner, and, thus, a violation of quality (Example 10) is expected.

8. U: What is the best car I can rent?
  S: Some experienced car drivers prefer Japanese cars to American cars. To rent a car, think of how you will use the car. Small cars are good for individual users, whereas bigger ones are the best choice for families. Some people like to rent Japanese cars. (People’s preference of Japanese cars has been repeated twice in the response.)
9. U: How can I achieve coins in X game?
  S: First, you need to kill the old man with a knife. (The sentence does not clearly indicate whether *with a knife* describes the man or how the action of killing should be done.)
10. U: Is it safe to take melatonin to induce sleep?
  S: Melatonin is safe if you do not become addicted to it.
  U: I used it for 5 days, but I only felt dizzy with no energy in the daytime.
  S: As an AI Chatbot, I cannot provide an answer. It would be better if you visit a specialist. (The Chatbot contradicts itself because in its earlier response it comments on the safety of melatonin.)

Regarding society-level errors, such errors are only interpreted with reference to the wider context, or more specifically, the social context. Hence, Chatbot responses may suggest some bias against one social group (Example 11) or lack politeness. Also, they can be simply unacceptable, or they lack common sense (Example 12; Higashinaka et al., 2015a, 2015b, 2021).

11. U: I am suffering from bullying in school. Help me avoid being bulled.
  S: If you are an *** or *** living in ******, think of imitating their accent when you use English in school (the Chatbot shows some bias against some ethnic groups).
12. People usually crawl between 7 and 12 months old. (The Chatbot should have used the word *babies* in place of *people* and, thus, its response lacks common sense.)

To determine whether or not a Chatbot’s response leads to a breakdown in communication, Higashinaka et al. (2015a, 2015b) suggested a classification of the types of communication breakdowns. According to Higashinaka et al. (2015a, 2015b), errors are considered, but they are not serious enough to cause a breakdown. Examples of such errors are grammatical errors, or those of repetition and society-based errors. On the other hand, some errors may lead to a possible breakdown because one cannot continue the conversation in a smooth manner. Errors of ignoring expectations and questions are examples. Other errors that may cause a possible breakdown include those of contradiction, ambiguous words, expressions, or common-sense errors. However, some errors are serious to the extent that they lead to a breakdown, and a conversationalist would find it difficult to continue the conversation (e.g., producing uninterpretable responses or those with wrong information).

## 3. Dataset Overview

Following previous researchers (e.g., Pérez-Rosas et al., 2018; Rashkin et al., 2018), we based our dataset on human–human conversations available online because clinical or real psychological counseling is of limited accessibility and availability (Das et al., 2022). Thus, the corpus of the psychological counsels is based on six counsels written in Saudi Arabic. They have been collected from a forum for mental health support available online called Nafsany https://www.nafsany.cc/vb/ (accessed on 9 September 2023). The forum is known among people suffering from various psychological issues. Forum users post their queries in the form of consultations, where other users, including patients, psychologists, or psychiatrists, respond to their queries in a thread. The forum is still popular among many current users because anonymity and confidentiality are ensured. Each user registers in the forum with a pseudonym. Patients with psychological problems feel that such forums are the only way to find another person with the same struggle, as, in some societies, complaining about a psychological disease is condemned and associated with a stigma. Hence, such social networking websites are much in demand nowadays.

Each consultation is a one-way interaction in a way similar to how people use conversational bots nowadays. Six forum users contributed to the present case study. Six conversations on six different psychological counsels (e.g., night terror, depression, social phobia, panic attacks, anhedonia, borderline personality disorder) constitute a corpus of 7245 tokens (i.e., words). Such psychological problems have been explored by many researchers who viewed them as the most common in clinical counselling (Atiyah, 2023; Liu et al., 2023). There are four-to-nine questions that require Chatbot responses in each consultation. The focus was on how ChatGPT-3.5 would respond to such queries in a cooperative manner. Thus, data analysis was restricted to ChatGPT’s responses. Each consultation resembles a casual conversation, and all the consultations start with a request for help and end with a thank-you statement. The corpus of ChatGPT’s responses is 6038 tokens, whereas that of queries is 1252 tokens. In general, the total number of queries is 43, and the total number of responses is 43. The average number of words for each ChatGPT’s response is 140 words, and for each query, it is 29 tokens.

## 4. Methodology

The researchers followed a qualitative, descriptive approach to examine to what extent ChatGPT-3.5 responds cooperatively to patients’ queries. The model of errors used is the one developed by Higashinaka et al. (2015a, 2015b, 2021) based on Gricean maxims. As stated above, the framework developed by Higashinaka et al. (2015a, 2015b, 2021) was chosen specifically because of its comprehensiveness. It is theory- and data-driven and encompasses linguistic, pragmatic, and social parameters. It is based on H. P. Grice’s (1975) theory of the Cooperative Principle and Schegloff and Sacks’s (1973) adjacency pairs. More importantly, the framework is based on data and errors typical of Chatbots. Moreover, Higashinaka et al. (2015a, 2015b, 2021) designed their framework taking into account that users interact with Chatbots, and, thus, the framework describes two-way interactions.

ChatGPT-3.5 was specifically selected for this study because it is the most commonly used model among Arabic-speaking users (Al-Khalifa et al., 2023), and it can produce responses that are close to humans’ (Z. Li et al., 2021). The present study is considered a case study, and, thus, it aims at carefully delineating the phenomenon (VanWynsberghe & Khan, 2007) of interacting with a Chatbot using ChatGPT-3.5. Since the focus of this study is on communication errors, an error is defined as the occurrence of an event that might negatively affect the flow of a conversation. Some errors can be serious, resulting in a communication breakdown, but others might not affect the quality of a conversation (Sanguinetti et al., 2020). It is important to note that the unit of analysis is the utterance that can be interpreted in relation to the wider context of society.

### 4.1. Procedure

**Figure 1:**
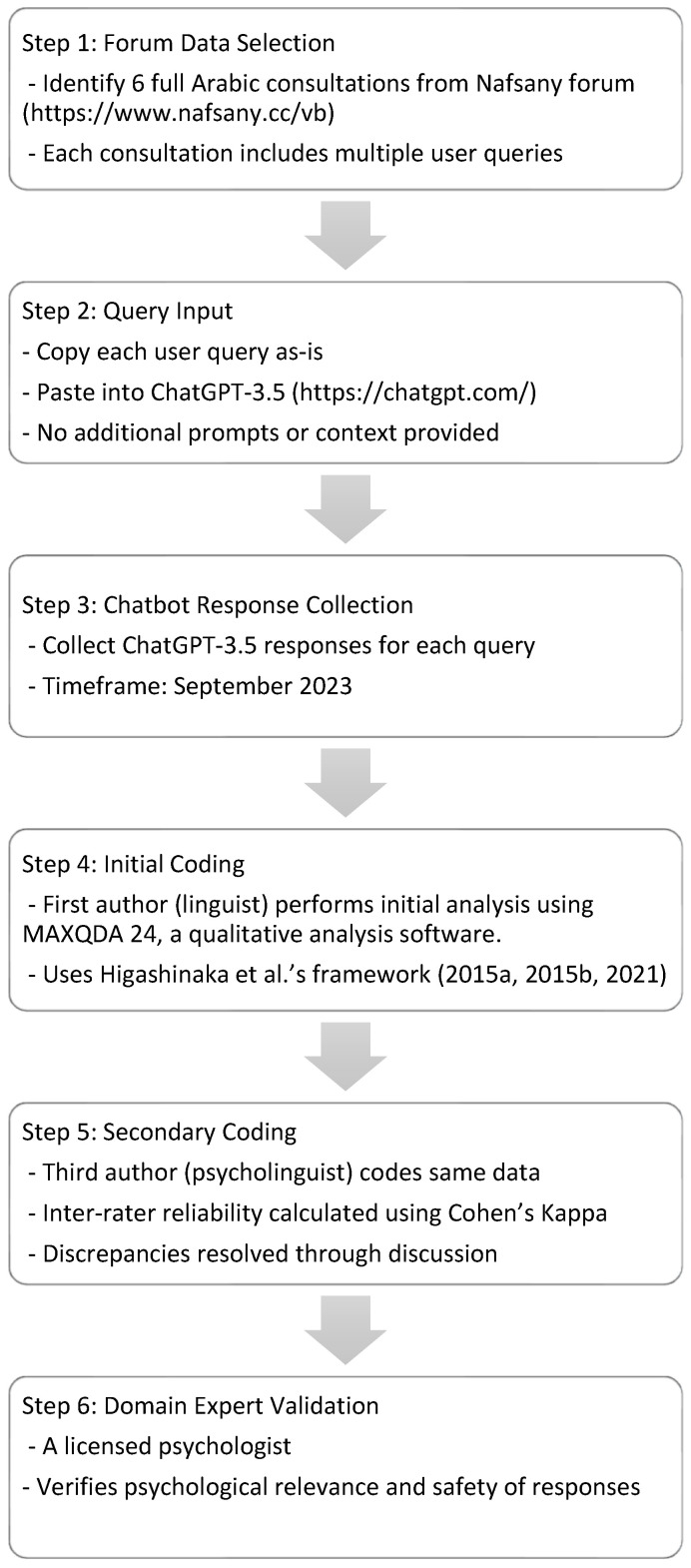
Study’s procedures (Higashinaka et al., 2015a, 2015b, 2021, framework).

The researchers followed a well-defined procedure to ensure that the resultant interactions are similar to how users utilize Chatbots in real life (see Figure 1).

To obtain responses to mental health–related queries, the second author, who is a professor in computer science, copied each user’s query in each consultation posted in the Forum and pasted it in ChatGPT-3.5 chat box https://chatgpt.com/ to obtain a Chatbot response. No specific instructions were provided to ChatGPT. It is important to note that ChatGPT-3.5 required a subscription, and the researcher turned off the Memory Feature so that the first consultation on a specific mental problem would not affect the Chatbot’s response to the second query. Data collection was initiated and completed in September 2023.

**Figure 2:**
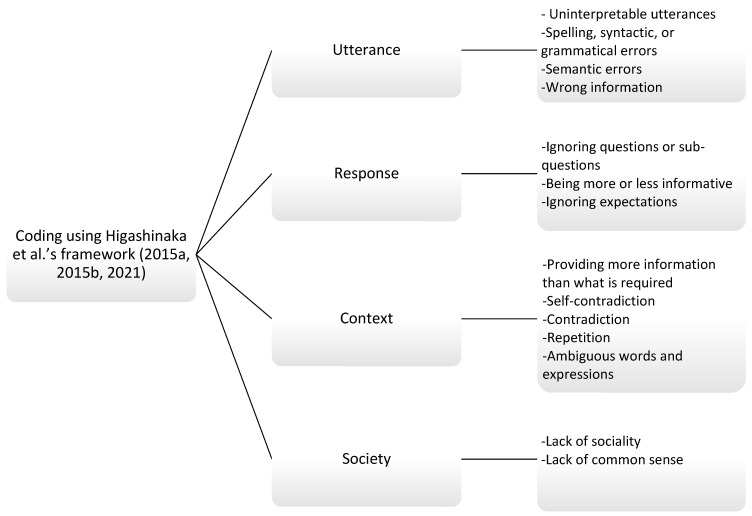
Error coding (Higashinaka et al., 2015a, 2015b, 2021, framework).

The data have been initially analyzed by the first author, who is a linguist with sufficient knowledge of pragmatics. The coding process was done systematically using Higashinaka et al.’s (2015a, 2015b, 2021) framework shown in Figure 2.

To verify data analysis, the third author, who is a psycholinguist, worked as the second coder of data. Further, the accuracy of the information provided by the Chatbot on mental health support was verified by a psychologist with 18 years of experience in psychotherapy and psychological counseling. The steps followed to collect and analyze data are outlined in Figure 1.

### 4.2. Analytical Software

**Figure 3:**
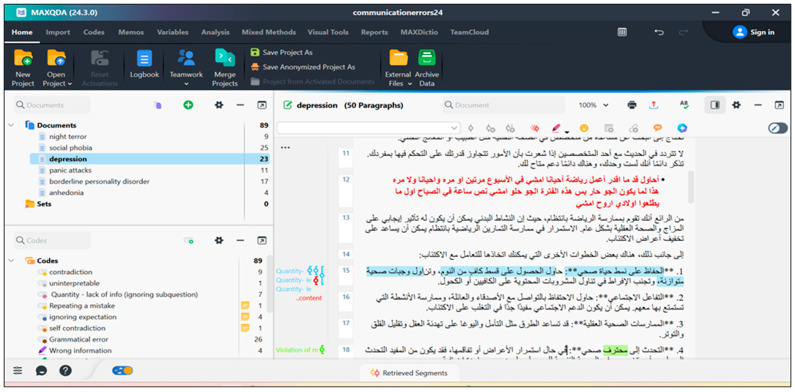
The qualitative analysis of Chatbot’s communication errors in a consultation about depression using MAXQDA 24.

MAXQDA 24, a qualitative analysis software (Atmane, 2024), was used to analyze Chatbot’s responses with respect to the categories listed above. The software helps in annotating communication errors by colorfully coding categories of errors and their subcategories (see Figure 3). By clicking on each category, one can include their comments and justify their code choice. More importantly, categories can be deactivated if one is not sure of including some categories in the framework above. The software has been used mainly by the first author. Then, WORD files of the six councils have been coded by the third author to achieve reliability in data analysis.

### 4.3. Reliability

To achieve inter-rater reliability (i.e., attaining consistency in the obtained results if coding is done by two raters; Krippendorff, 2004), the first author initially coded the data using MAXQDA 24. Then, the third researcher coded the same data using Word Highlight Feature. The third author did a sample analysis first to ensure that any initial discrepancies between the two coders had been resolved. The first rater identified 88 errors, whereas the second rater found about 100 communication errors.

The disagreement between coders was on whether self-contradiction is a separate category or subsumed under that of contradiction. Further, uninterpretable utterances, such as tawtiːr wa furdʒat al-ʕadˁalæt (توتير وفرجة العضلات; see Table S1 in the Supplementary Materials), have been coded with those of manner by both coders. More importantly, when the patient used a misspelt Arabic name (i.e., أولتابروا əʊltaːbrəʊ in place of إسيتالوبرام estaləʊbraːm) of the medicine “Escitalopram,” the Chatbot repeats the misspelt name as أولتابرا əʊltaːbraː. This is coded as part of the category of spelling and grammatical errors. More notably, major discrepancies between the first and the second coder were mainly on instances of repetition, ignoring expectations, and syntactic, spelling, and grammatical errors. As the first coder did not count repetitive examples of spelling, language, and grammatical mistakes, the second coder included such repetitions (i.e., the wrong word choice in the use of muħtarif محترف “professional” as a direct translation of professionals offering psychological counseling). In addition, the second coder included five more examples of ignoring expectations, which have been regarded as instances of contradiction by the first rater. To resolve this discrepancy, contradictory examples have been reserved for Chatbot responses that contradicted those of the user, whereas violations of users’ expectations were kept for elaborations by the Chatbot based on input queries. For example, the Chatbot included in its responses the reference to a male physician “tˤa.biːb,” though the input query has a mention of a female physician “tˤa.biː.bah.” This is marked as an example of a contradiction. More notably, the second rater added more examples of repetition than the first rater. Such examples pertain mainly to the use of mediation, breathing and debriefing techniques, exercising, etc.

The researchers have used Cohen’s kappa to measure inter-rater reliability. Cohen’s kappa would eliminate any chance agreement where the two coders might by chance have provided the same code to the same category (McHugh, 2012). The website https://www.graphpad.com/quickcalcs/kappa1/?k=11 (accessed on 10 June 2025) has been utilized to calculate Cohen’s Kappa, where data were inserted in 11 categories since the two coders did not find examples of semantic errors or those related to quantity. Cohen’s kappa of 0.77 indicates substantial agreement between the two raters.

## 5. Results

Interacting with ChatGPT-3.5 to provide psychological consultations resulted in 102 communication errors. Utterances with grammatical errors are more common than others. The category of grammatical errors was ranked first with 34 occurrences (33.33%), followed by that of repetition (i.e., 30 errors; 29.41%), where the Chatbot provides less information, but in the form of paraphrases and repetitions (see Table 2). However, uninterpretable utterances and self-contradictions are the least frequent errors, resulting in only one example for each (i.e., 0.98%), followed by responses lacking in common sense (i.e., 1.96%), others with wrong information (i.e., 3.92%), or those ignoring expectations (i.e., 3.92%).

As shown above, grammatical and syntactic errors, including spelling mistakes, occur most frequently. Examples of such errors include those of verb–subject agreement in gender in Arabic, as in tuːɁθr kul nəʊʕ (تؤثر كل نوع) instead of juːɁθr kul nəʊʕ (يؤثر كل نوع), which are translated as “each type affects.” Apparently, the system confuses gender for nouns that are not inherently feminine, and, thus, they are treated as masculine, and the opposite is true for nouns that are inherently or biologically not masculine (i.e., nəʊʕ “type”). In Arabic, there is the category of grammatical gender, where nouns need to be marked for gender, though they do not naturally show masculine or feminine traits. However, feminine nouns are morphologically marked, whereas masculine nouns are unmarked (Alkohlani, 2016). The Chatbot incorrectly uses a masculine verb, as in qad yaxtalif alistidʒaːbah (قد يختلف الاستجابة “response may differ”), despite the subsequent noun carrying a feminine suffix (e.g., alistidʒaːbah “response”). Other examples of gender confusion are ta.naː.wulu wa.dʒa.baːt qad tu.xaf.fif (تناول وجبات قد تخفف “eating meals may lessen”) instead of ta.naː.wulu wa.dʒa.baːt qad ju.xaf.fif (تناول وجبات قد يخفف) where wa.dʒa.baːt “meals” is marked feminine, but ta.naː.wul “eating” is a masculine verb, and, thus, the verb should denote a masculine subject as in ju.xaf.fif “lessen.” Spelling mistakes, on the other hand, are mainly attributed to the fact that some words are confused with others because they provide nearly the same meaning. The system’s used words are not typically utilized in such contexts. However, some of the words used in the responses may provoke a negative meaning, as in using al.waḍ.ʕij.ja (الوضعية) in at.ta.ʕaː.mul ma.ʕa haː.ði.hi l.waḍ.ʕij.ja (التعامل مع هذه الوضعية) in place of al.waḍʕ (الوضع), in which the former means “dealing with this pose” and the latter means “dealing with this situation.” In other words, the word al.waḍ.ʕij.ja (الوضعية) means “pose,” whereas the al.waḍʕ (الوضع) denotes the meaning of “situation” or “case.”

As for errors of repetition, which occupied the second place with 30 instances, they mainly comprise relaxation and breathing techniques, following a healthy lifestyle and physician’s instructions on the recommended dosage, meditating, eating healthy food, sleeping well, and taking sessions of cognitive behavioral therapy (see Table S1 in the Supplementary Materials). On the other hand, errors of contradiction placed third with nine examples (i.e., 8.82%), and they are mainly in reference to a male physician instead of a female, as some users mentioned that they have visited a female physician. Another instance of contradiction is when the Chatbot provides advice on cooperating with school staff, where the user refers to the patient at the college level. However, other frequent context-related errors are those of using ambiguous words and expressions by the Chatbot, and there are four examples (i.e., 3.92%). One notable example of ambiguity is the advice on avoiding heavy meals to lessen the effect of panic attacks. The ambiguous word *heavy* is provided without any definition or examples of such meals. It can provide the meaning of *fatty*, *dairy*, or those with beans, or others that make you feel full. All of such may disturb the digestive system and trigger panic attacks in some patients.

Other less common errors include ignoring questions or sub-questions or lacking in sociality, and they ranked fourth and fifth with seven (6.86%) and six (i.e., 5.88%) instances, respectively. One example of the former is when the user mentioned in the question that they have increased the dose of sertraline and that it did not relieve the symptoms. In another consultation on borderline personality disorder, the caregiver reported that the patient can be easily irritated and that they wanted to go out with friends, but their friendship does not last. Nevertheless, the Chatbot does not respond to such sub-questions, indicating a violation of the quantity maxim. Regarding the criterion of sociality, one notable example of an utterance that shows some lack of respect is the advice to avoid alcohol provided to Saudi women, as it is common that Saudi women basically do not drink alcohol, and alcohol consumption is prohibited in the Kingdom (see Table S1 in Supplementary Materials). One more example is the Chatbot’s wish for a user to submit themselves to God and accept the situation. Apparently, this response lacks politeness.

أتمنى لك الصحة والعافية دائمًا، وأن تجد الراحة والتسليم بما هو خير لك.

I always wish you good health, and that you find comfort and peace and submit to God whatever the best for you.

More of the least-common communication errors include providing wrong information and ignoring expectations, where there are four (i.e., 3.92%) examples for each. An instance of wrong information is the Chatbot’s response that if one stops taking antidepressants suddenly, it may make one prone to psychosis. This example has been repeated in the same consultation. The error is mainly due to some shortage in the training data, where ʕaq.liː (عقلي), in place of naf.siː (نفسي), has been provided as a direct translation of “mental.” In Arabic psychology, the former is employed to describe symptoms of psychosis such as hallucinations and delusions, whereas the latter is used to describe anxiety, depression, etc. However, in English, the word “mental” is used for both. Further, in one consultation, the Chatbot mentions stress and emotional stress as examples of stimulants besides coffee and tea. As for ignoring expectations, in one response, the Chatbot refers to the patient’s attempts to treat themselves, though the patients themselves have not mentioned any. Moreover, the Chatbot advises the caregiver to visit a pediatrician, although the caregiver has mentioned earlier that the patient is at college level (see Table S1 in Supplementary Materials).

The least-common Chatbot errors include nonsensical or uninterpretable utterances, or other self-contradictory responses. Examples of an utterance that does not make sense is when the Chatbot refers to sleeping time as the time when anxious people are pressurized the most. One more instance is when the Chatbot mentioned that birth control pills can differently affect individuals, but part of common knowledge, as we know, is the fact that only women can be pregnant (see Table S1 in Supplementary Materials). As for uninterpretable utterances with nonsensical words, one notable example is tawtiːr wa furdʒat al-ʕadˁalæt (توتير وفرجة العضلات) after a sentence on muscle relaxation. The two words tawtiːr and furdʒat contribute no meaning in Arabic, and, thus, they are nonsensical, except for the word al-ʕadˁalæt “muscles.” For self-contradictory responses, in only one instance, the Chatbot mentioned that it cannot offer any medical advice because it is an AI model developed by OpenAI.

هل تنصحني بالتوقف عنها؟

كوني أنا موديل لغة اصطناعية تم تطويرها بواسطة أوبن إي آي، فإنني لا أستطيع تقديم النصائح الطبية الشخصية. ومع ذلك، يمكنني تقديم بعض النصائح العامة.

Do you advise me to stop taking them?

As an artificial language model developed by OpenAI, I cannot provide personalized medical advice. However, I can offer some general advice.

However, it provided two pieces of advice in its earlier responses to questions about whether or not birth control pills cause depression, as well as the use of Escitalopram to treat depression.

To answer the second research question on whether or not such errors cause a communication breakdown, Table 3 illustrates the percentages of errors that cause a possible or a serious breakdown.

As shown above, errors hindering smooth communication with a Chatbot do not constitute more than 5%. Next, in terms of severity, are errors that may trigger a possible breakdown, and they make up less than one-third of the total. On the other hand, about two-thirds (i.e., 68.6%) of the errors do not cause a communication breakdown. Results clearly indicate that ChatGPT-3.5 is capable of interacting effectively with humans, even in emotionally loaded interactions, such as providing psychological consultations on various psychological issues.

## 6. Discussion

The present study aimed to answer the following research questions: What types and patterns of communication errors occur in interactions between humans and ChatGPT-3.5 in Arabic, and how do these errors impact the effectiveness of AI-based mental health support?

Results revealed that using ChatGPT-3.5 in mental health support might not result in a communication breakdown, as the majority of errors are mainly repetitions or spelling or grammatical mistakes. Previous researchers (Lissak et al., 2024; Sanguinetti et al., 2020) note that redundancy or repetition is typical of conversational Chatbots. As illustrated by the results, advice on changing lifestyle, meditating, doing relaxation techniques, and following instructions on the recommended dose has been repeated in almost every session. Though ChatGPT-3.5 is the closest to human responses (Z. Li et al., 2021), Tsvilodub et al. (2023) argue that some neural models fail to include the right subset of information that fits into a specific context or reflects an individual’s experience, and, thus, the Chatbot chooses to include all potential information. Hence, as reported by B. Wang et al. (2023), repetition is expected from ChatGPT-3.5. Nevertheless, repetition is highly recommended in psychological counselling, especially in the domain of psychodynamic psychotherapy, where it proves to be beneficial to treat depression (Corradi, 2009). This will eventually improve users’ satisfaction and achieve some essential level of assurance and tranquility. However, excessive or uncontextualized repetition might also be perceived as impersonal or mechanical, especially when the user seeks individualized or culturally sensitive guidance. Such perception could reduce user engagement and trust in the tool, as repetition in this case may reflect limited understanding of user-specific needs rather than therapeutic reinforcement.

On the other hand, the frequency of linguistic errors is attributed to the fact that ChaGPT 3.5 is trained on a limited number of data that lacks linguistic diversity (Meier et al., 2024). Thus, the Chatbot confuses words that are orthographically similar or provide nearly the same meaning. For example, in psychological discourse, tan.fiːs (تنفيس) is more frequently used in psychological counsels to refer to psychological debriefing and, thus, the confusion between tan.fiːs (تنفيس) to refer to debriefing and ta.naf.fus (تنفس) for breathing in air. Other common errors include those of verb–subject agreements where the Arabic verb refers to a female subject, but the collocating subject denotes a male doer. The opposite is true for female doers. This happens only for nouns that belong to the category of grammatical gender in Arabic. Such errors, while simply grammatical, may have deeper socio-cultural implications for Arabic-speaking users, where gender distinctions in language are closely tied to social identity and respect. Misgendering through verb agreement, especially in sensitive contexts like mental health counseling, can be perceived as dismissive or disrespectful. Apparently, gender mismatch is caused by some shortage in the training data and some flaws in translation. Though Arabic is not a sexist language, gender mismatch between verbs and nouns might suggest some discrimination against female users, as the Chatbot favors masculine verbs over feminine verbs. As Middle Eastern societies are inherently patriarchal, resorting to a Chatbot that shows some sexism may discourage female users from using the model for mental health queries. Using sexist language not only risks reducing the perceived professionalism of the AI model but may also undermine user trust, as gendered language is often used to affirm social roles, values, and relational dynamics.

In addition, the analysis showed errors of wrong word choice, which might be the result of direct translation of some English information (Qian et al., 2023), such as the reference to professionals as muħ.ta.rif محترف “professional,” which is awkward in Arabic psychological discourse. Apparently, as emphasized by Alanezi (2024), AI conversational bots are not trained to understand the different nuances of languages, besides the fact that Arabic is a low-resource language. More notably, in this study, users use their colloquial language or dialect, which makes it difficult for bots to understand content and respond adequately and accordingly. Such misinterpretations, particularly in therapeutic contexts, could negatively affect user confidence in the Chatbot’s capability. Mistranslations or gender mismatches not only reduce linguistic precision but may also signal to users a lack of awareness of their cultural or personal identity, leading to diminished trust and willingness to engage with the Chatbot.

Results also reveal that other less frequent errors include responses contradicting the user’s, as well as ignoring questions or sub-questions and sociality errors. This is consistent with findings of Sanguinetti et al. (2020), who reported that ignoring questions is one of the four common errors in Chatbot responses, besides repetition, and of Barattieri di San Pietro et al. (2023), who found that the maxim of quantity, corresponding to repetition and ignoring questions and sub-questions, is one weakness in the Chatbot’s responses. Similarly, Barattieri di San Pietro et al. (2023) further argue that the quantity maxim is difficult to manage by Chatbots. However, Z. Li et al. (2021) state that contradiction is one serious problem of dialogue systems because it may eventually lead Chatbot users to lose trust and discontinue the conversation. From a user’s perspective, violating the quality maxim and being inconsistent may make one think of whether or not Chatbots understand what they are saying (Nie et al., 2020). These findings indicate that even if such errors are infrequent, they may carry disproportionate weight in shaping user perception, especially in sensitive contexts like mental health. A single contradiction or ignored question could undermine rapport and provide the impression that the Chatbot is inattentive or unempathetic. This may be particularly problematic for vulnerable users seeking emotional support.

As for responses lacking sociality, there are differences between cultures in terms of what is considered polite and what is not (Yin et al., 2024). For a Saudi female, the advice on reducing alcohol consumption is socially impolite, but for users from other cultures, the response does not lack politeness. Alanezi (2024) and Ajlouni et al. (2023) show that ChatGPTs are not adapted to reflect various cultural beliefs. More importantly, because Chatbots are trained more on specific data drawn from particular cultures, they can show some inherent bias (Danks & London, 2017; D’Silva & Pande, 2024; Yin et al., 2024). In addition, Danks and London (2017) elaborate that if data do not comprehensively describe different populations, Chatbots might produce some inevitable bias. More notably, Kalam et al. (2024) mention that ChatGPTs cannot customize responses to individual users and their concerns. Thus, they may provide inappropriate or overgeneralized recommendations (Alanezi, 2024). Further, Yin et al. (2024) note that some cultural biases can be very serious, leading to deterioration in Chatbot performance. Hence, ensuring a polite relationship between the user and the Chatbot helps in establishing trust and cooperation (Lee & Lee, 2022). This supports the need for developing AI systems with culturally adaptive capabilities that meet users’ beliefs and expectations. In high-context cultures such as many Arabic-speaking societies, politeness is integral to communication. Misalignment in such areas can lead users to perceive AI as insensitive or disrespectful, threatening its adoption and effectiveness.

Other communication errors that are less common than the ones discussed above are those of the provision of wrong information, ignoring expectations, and others with utterances with ambiguous words. Ignoring expectations is pertinent to contradictions because when humans interact with Chatbots, they want them to meet their expectations, but resultant contradictions disrupt communication flow (Nie et al., 2020). Sanguinetti et al. (2020) claim that Chatbots’ failure to meet expectations might result in frustration. As for providing fabricated information as an example of a quality maxim violation, it has been confirmed by Currie (2023), Garg and Chauhan (2024), and Gravel et al. (2023), who argue that ChatGPT-3.5 is susceptible to factual errors and, thus, integrating ChatGPT-3.5 in clinical practice and manuscript writing may affect one’s level of integrity and professionalism. Further, in terms of reliability concerns, Alanezi (2024) notes that one of the interviewees, who used ChatGPT for mental health support, wrote that ChatGPT provides inaccurate or incomplete information. Even worse, D’Silva and Pande (2024) and Oviedo-Trespalacios et al. (2023) emphasize that ChatGPT might provide unreliable data and harmful information. However, for the inclusion of ambiguity in responses, Sanguinetti et al. (2020) observed that manner maxim violations, in general, were the least likely to occur in Chatbot responses compared to other maxims of quantity and relation.

Results also indicate that the least occurring errors include self-contradiction, common-sense violations, and uninterpretable responses. For self-contradictory responses, though they occurred only once, they are typical of many AI Chatbots when they provide some information on sensitive topics or refuse to answer questions because of a lack of information (Blum, 2022). Blum (2022) notes that ChatGPT will not proceed to answer a question even if it can prioritize safety. Thus, the category of self-contradictions is not as common as contradictions. As for common-sense violations, ChatGPT, though displaying human-like reasoning capabilities, falls short in terms of its understanding of some nuances, especially in contexts of specific cultures or languages (McIntosh et al., 2024). Moreover, as a sub-category of the manner maxim and as reflected by the results, uninterpretable responses are the least likely to occur, as confirmed by Sanguinetti et al. (2020).

The above discussion illustrates that ChatGPT-3.5 has the potential to provide effective mental health support because the serious errors that might cause a communication breakdown do not constitute more than 5% of the total errors found in the responses. More importantly, errors in providing wrong information might be the result of direct translation between English and Arabic. Hence, the model tends to offer the same advice no matter who the patient is and from which culture (Kalam et al., 2024). For example, the use of the Arabic word ʕaqli:jah (عقلية) as a translation of “mental” to refer to disorders strictly means psychosis, and it is a direct translation between English and Arabic. On the other hand, the majority of errors are mainly repetition, linguistic errors, and sociality. As mentioned above, repetition is sometimes recommended in psychotherapy as some patients need to continuously hear reassuring statements (Corradi, 2009). Additionally, sociality errors that might result from interacting with ChatGPT-3.5 are repeated in almost every conversation, which suggests that though ChatGPT-3.5 is capable of producing humanlike responses, it needs to be sensitive to cultural differences, especially if one uses their dialect to interact with the model.

### Limitations, Practical Implications, and Future Research Directions

Despite the valuable insights yielded by this study, several limitations must be acknowledged. First, the dataset comprised only six Arabic-language consultations, which is relatively small. A larger and more diverse corpus is needed to gain a more comprehensive understanding of the challenges associated with AI-based mental health support interactions. Moreover, the study focused exclusively on a single AI system—ChatGPT-3.5. Future research should explore and compare the performance of alternative systems, such as Google Gemini, DeepSeek, ChatGPT-4o, and Microsoft Copilot, to provide a broader assessment of AI capabilities in this domain. Another limitation relates to the absence of user evaluations. Incorporating user feedback and perceptions in future studies would provide valuable insights and enhance our understanding of the impact of communication errors on user experience.

Additionally, future research should consider the influence of variation in Arabic dialects because this may affect both the nature of interactions with AI systems and the linguistic appropriateness of AI-generated responses. Another important factor is the length of user queries, which may significantly influence the linguistic quality of the AI’s responses. Excessively short or overly long inputs may hinder the model’s ability to produce coherent and contextually appropriate responses, potentially leading to vague, incomplete, or grammatically flawed outputs. This introduces variability in response quality and complicates the consistent evaluation of linguistic performance. The findings of this study, including the types of errors identified in ChatGPT’s output, should inform the future development of AI systems to improve their linguistic and contextual reliability.

Finally, the results of the current study will enable users to understand how their input language shapes the quality of AI-generated responses. Familiarity with effective input strategies may help users receive more linguistically accurate, relevant, and meaningful support.

## 7. Conclusions

In the present study, we examined the common communication errors in ChatGPT-3.5’s interactions within the context of mental health support in Arabic. A corpus of 7245 tokens based on six Arabic-language consultations was collected from an online mental health support forum. For each consultation, the researchers submitted the users’ Arabic queries to ChatGPT-3.5 and analyzed the system’s responses using the model of Higashinaka et al. (2015a, 2015b, 2021). The findings revealed that ChatGPT-3.5 demonstrated promising capabilities, and the interactions were relatively human-like, with communication errors that are mostly grammatical and repetitive. However, such errors did not cause a breakdown in communication.

However, the analysis also highlighted several challenges in capturing cultural and linguistic nuances, particularly when users interacted using regional dialects. A small number of misinformation instances were also detected, raising concerns about the system’s reliability in unsupervised mental health contexts. These errors, although not always frequent, may impact user trust and the perceived credibility of the tool, especially in high-stakes or culturally sensitive conversations. The study addressed its research question by showing that while most errors were minor (e.g., repetitions or grammatical issues), others—such as contradictions or inappropriate advice—could disrupt communication or lead to user disengagement. These findings highlight the importance of developing AI systems that are not only linguistically competent but also culturally aware. Due to the novelty of AI language models in this field, further research is needed to evaluate their communicative behavior in diverse languages and mental health scenarios. Developers of such systems should diversify training data, improve cultural adaptation, and incorporate safeguards for safe, context-aware interactions. Above all, AI tools like ChatGPT should be viewed as supportive—not substitutive—resources and must be used responsibly under appropriate human oversight, particularly in mental health care.

## Figures and Tables

**Table 1:** Communication errors in Chatbot–human interactions as suggested by Higashinaka et al. (2015a, 2015b, 2021).

Dialogue Parameters	Sub-Categories of Error Types
Utterance	(1) Uninterpretable utterances
	(2) Syntactic or grammatical errors
	(3) Semantic errors
	(4) Wrong information
Response	(5) Ignoring questions
	(6) Being more or less informative
	(7) Ignoring expectations
Context	(8) Providing more information than what is required
	(9) Self-contradiction
	(10) Contradiction
	(11) Repetition
	(12) Ambiguous words and expressions
Society	(13) Lack of sociality
	(14) Lack of common sense

**Table 2:** The distribution and number of communication errors found in psychological consultations provided by ChatGPT-3.5.

Dialogue Parameters	Type of Error	Frequency of Errors	Percentage
Utterance	(1) Uninterpretable utterances	1	0.98%
	(2) Spelling, syntactic, or grammatical errors	34	33.33%
	(3) Semantic errors	-	0.00%
	(4) Wrong information	4	3.92%
Response	(5) Ignoring questions or sub-questions	7	6.86%
	(6) Being more or less informative	-	0.00%
	(7) Ignoring expectations	4	3.92%
Context	(8) Providing more information than what is required	-	0.00%
	(9) Self-contradiction	1	0.98%
	(10) Contradiction	9	8.82%
	(11) Repetition	30	29.41%
	(12) Ambiguous words and expressions	4	3.92%
Society	(13) Lack of sociality	6	5.88%
	(14) Lack of common sense	2	1.96%
Total		102	100%

**Table 3:** The distribution and number of errors with respect to types of communication breakdowns.

Category of Errors	Number of Errors	Percentage
Not a breakdown	70	68.60%
Grammatical errors		
Repetitions		
Sociality errors		
Possible breakdown	27	26.40%
Ignoring expectation		
Ignoring questions or sub-questions		
Contradiction		
Self-contradiction		
Common-sense errors		
Ambiguous words and expressions		
Breakdown	5	4.90%
Uninterpretable responses		
Wrong information		
Total	102	100%

## Data Availability

Data will be made available on request.

## Supplementary Materials

The following are available online at https://www.mdpi.com/article/10.3390/bs15081119/s1, Table S1: Examples of communication errors provided by ChatGPT-3.5 in its responses to mental health queries.
