# Supplementary material for: Communication Errors in Human–Chatbot Interactions: A Case Study of ChatGPT Arabic Mental Health Support Inquiries
Source: Behav Sci (Basel). 2025 Aug 18;15(8):1119. doi: 10.3390/bs15081119 (PMC12382934; doi:10.3390/bs15081119)
Supplement: Supplementary file 1 [file behavsci-15-01119-s001.zip › behavsci-3679644-supplementary.pdf]

behavioral sciences

MDPI

Supplementary Materials

Table S1. Examples of communication errors provided by ChatGPT-3.5 in its responses to mental health queries.

Type of Error	Example	Transcription/Translation
(1) Uninterpretable utterances	جي بي تي: *توتير وفرجة* العضلات	ChatGPT: tawtiːr wa furdʒat al-ʕadˤalæt<br>……………………….muscles
(2) Spelling, syntactic, or grammatical errors	verb-subject agreement in gender<br>جي بي تي: ومع ذلك، يجب أن نلاحظ أن العديد من العوامل يمكن أن تسهم في الاكتئاب، ولا يمكن تحديد العامل الفردي الذي يسبب الاكتئاب بالضبط. بالإضافة إلى ذلك، هناك أنواع مختلفة من وسائل منع الحمل، وقد *تؤثر كل نوع* بشكل مختلف على الأفراد	tuːʔθr kul nəʊʕ in place of juːʔθr kul nəʊʕ<br>ChatGPT: *Each type affects*…
	adding or deleting letters or replacing them<br>المستخدم :سيكون عندنا موعد مع الاخصائية غدا وقبل الموعد بساعة أريد ان اكتب لها بآخر التطورات التي صارت<br>جي بي تي :الهدف الرئيسي هو التأكد من أن أي قرار تتخذوه يكون مبنيًا على مصلحة ابنتك وصحتها النفسية. لا تترددي في طرح هذه الأسئلة والأفكار مع الاختصاصية والتعاون معها للعثور على *الحلا* للمشكلة.	User: We have an appointment with the specialist tomorrow, and an hour before the appointment, I want to write to her about the latest developments.<br>ChatGPT: The main goal is to ensure that any decision you make is based on your daughter's best interests and her mental well-being. Don't hesitate to raise these questions and ideas with the specialist and collaborate with her to find (*dessert*) to the problem. (al.ħa.laː 'الحلا', which means "dessert," is used in place of al-ħall (i.e., solution. This is due to the addition of a letter)
	wrong word choice<br>المستخدم: عطوني طقوسكم قبل الذهاب لمثل هذه المناسبات و لا تبخلون علي باي نصيحة<br>جي بي تي :قم بوضع خطة بسيطة لما ستفعله أثناء المناسبة، مما يمكن أن يجعلك *أكثر واثقًا*.<br>المستخدم :عندي مناسبة بعد كم يوم ومن وقت الدعوة الى الان وانا في حالة ما يعلم فيها الا الله حتى جهازي	User: Give me your rituals before going to such events, and don't hold back on any advice.<br>ChatGPT: Create a simple plan for what you'll do during the event, which can make you more confident. (The collocation ak.θar waː.θi.qan أكثر واثقاً

الهضمي انهار تماما من الخوف يعني معدة حموضة و قولون و ادخل دورة المياه تقريبا كل ساعة اعزكم الله	is used in place of ak.θar θɪ.qah أكثر ثقة. The word ak.θar collocates with θɪ.qah (i.e., more confident) in Arabic but not waː.θi.qan).
جي بي تي :التنفس والاسترخاء: حاول *تنفيس* الهواء ببطء وعميقًا عندما تشعر بالقلق. جلسة قصيرة من التأمل أو اليوغا يمكن أن تساعد في تهدئة الأعصاب.	User: I have an event in a few days, and from the time of the invitation until now, I've been in a state that only God knows. Even my digestive system has completely collapsed from fear—I'm dealing with stomach acid, IBS, and I go to the bathroom almost every hour, bless you.<br>ChatGPT: Breathing and relaxation: Try *debriefing* air slowly and deeply when you feel anxious. A short session of meditation or yoga can help calm your nerves. (The phrase tan.fiːs al.ha.waːʔ تنفيس الهواء is used instead of ta.naf.fus al.ha.waːʔ تنفس الهواء, in which the latter means "breathing in air." The word tan.fiːs refers to the debriefing technique and, thus, the confusion).
المستخدم: هي دائما ما تشتكي بان هناك من لا يفهمها<br>جي بي تي: إنه من المهم أن تعمل مع الاختصاصية النفسية على فهم تحديات ابنتك والأسباب التي تجعلها تشعر بأن هناك من لا يفهمها. قد يكون هذا جزءًا من التحديات التي تواجهها بسبب اضطراب الشخصية الحدية الذي تعاني منه. الاختصاصية يمكنها مساعدتك في فهم كيفية التعامل مع هذه *الوضعية* وتوجيهك بشكل مناسب حول كيفية دعم ابنتك.	User: She always complains that no one understands her.<br>ChatGPT: It's important to work with the psychological specialist to understand your daughter's challenges and the reasons she feels misunderstood. This might be part of the difficulties she faces due to the borderline personality disorder she suffers from. The specialist can help you understand how to deal with this *pose* and guide you appropriately on how to support your daughter. (al.waḍ.ʕij.ja الوضعية "pose" is used in place of al.waḍʕ الوضع "situation")
المستخدم: عندما أقول لها أني أحبك أنت من دمي ولحمي لا تصدق<br>جي بي تي: قد تكون ابنتك تعيش في مرحلة من التحديات و*التغييرات* النفسية التي تجعلها تشك في مشاعر الآخرين تجاهها، حتى إذا كانت تجرب مشاعر الحب والدعم منك. هذا أمر طبيعي في بعض الأحيان، ويمكن أن يحدث مع المراهقين.	User: When I tell her I love her, that she's my flesh and blood, she doesn't believe it.<br>ChatGPT: Your daughter might be going through a period of psychological challenges and *changes* that make her doubt others' feelings towards her, even when she's experiencing love and support from you. This is sometimes normal and can
syntactic problems<br>المستخدم: رفعت جرعة لوسترال فترة الى 100 وما حسيت بالفرق لكن يبدو أني سأجرب مرة ثانية بس بعد استشارة الطبيب لكن حتى اتخطا مناسبة الغد بما تنصحني واذا كنت في موقف مشابه وش تسوي بالضبط	

	المشكلة المناسبة بكرة ومافي وقت للحلول طويلة المدى<br><br>جي بي تي: لا تتردد في اللجوء إلى أي من هذه الطرق التي تعمل بشكل جيد بالنسبة لك. في النهاية، حاول أن تكون لطيفًا مع نفسك *وتقدم قدر الإمكان من الراحة* والدعم لنفسك في هذه اللحظة.	happen with teenagers. (ta.ɣiː.raːt تغييرات is used in place of ta.ɣaj.ju.raːt تغيّرات in which both mean "changes")<br><br>User: I increased my Lustral dose to 100 for a period and didn't feel a difference, but it seems I'll try again after consulting the doctor. However, to get through tomorrow's event, what do you advise me to do, and if you were in a similar situation, what exactly would you do? The problem is the event is tomorrow, and there's no time for long-term solutions.<br>ChatGPT: Don't hesitate to resort to any of these methods that work well for you. Ultimately, try to be kind to yourself and *offer yourself as much comfort and support as possible* in this moment. (The syntactic problem stems from the unnecessary addition of prepositions such as min من "from" as in wa tu.qad.dim qad.ra l.im.kaːn mi.na r.raː.ħa 'وتقدم قدر الإمكان من الراحة' in place of wa tu.qad.dim qad.ra l.im.kaːn ar.raː.ħa 'وتقدم قدر الإمكان الراحة' in which the sentence means "and provide as much comfort as possible")
(3) Semantic errors	-	-
(4) Wrong information	جي بي تي: قد يفيد أيضًا تجنب *المنبهات* مثل الكافيين *والتوتر والتوتر العاطفي*.	ChatGPT: It may also be helpful to avoid *stimulants* such as caffeine, *stress, and emotional tension*.
(5) Ignoring questions or sub-questions	المستخدم: *رفعت جرعة لوسترال* فترة الى 100 وما حسيت بالفرق لكن يبدو أني سأجرب مرة ثانية بس بعد استشارة الطبيب<br>لكن حتى اتخطا مناسبة الغد بما تنصحني واذا كنت في موقف مشابه وش تسوي بالضبط<br>المشكلة المناسبة بكرة ومافي وقت للحلول طويلة المدى<br>جي بي تي: إذا كانت المناسبة غدًا وتحتاج إلى حلول سريعة للتعامل مع القلق والتوتر، فإليك بعض النصائح العملية:<br>استخدم طرق التهدئة السريعة:	User: I *increased my Lustral* dose to 100 for a while, but I didn't feel a difference. It seems like I'll try again, but only after consulting the doctor.<br>But to get through tomorrow's event, what do you advise me to do? And if you were in a similar situation, what exactly would you do?<br>The problem is the event is tomorrow, and there's no time for long-term solutions.<br>ChatGPT: If the event is tomorrow and you need quick solutions to deal with anxiety and stress, here are some practical tips:<br>Quick Calming Techniques:

	قبل المناسبة، جرب تقنيات التنفس العميق والتأمل البسيطة للتهدئة وتخفيف القلق.<br>يمكن أن تكون تمارين الاسترخاء العضلي مفيدة أيضًا، حيث تركز على توتير وفرجة العضلات بشكل متسلسل.<br>تفكير إيجابي وتوجيه الأفكار:<br>حاول استبدال الأفكار السلبية بأفكار إيجابية. قل لنفسك أنك تستطيع التعامل مع هذه المواقف بنجاح.<br>قد يكون من المفيد تكرار التوجيهات الإيجابية لنفسك، مثل "أنا قوي وقادر على التغلب على هذا القلق".<br>التحدث مع شخص موثوق:<br>إذا كان بإمكانك التحدث مع شخص موثوق به قبل المناسبة، فهذا قد يساعد في تخفيف الضغط. شارك مشاعرك ولا تتردد في طلب الدعم.<br>استخدم التقنيات الموجهة للحدث:<br>قبل المناسبة، جرب استخدام تقنيات التوجيه للحدث.<br>تخيل نفسك وأنت تتعامل بنجاح مع الموقف المحرج أو المجهد.<br>الاستفادة من الدعم الدوائي:<br>إذا كنت تستخدم دواءً للتحكم في القلق، فتأكد من اتباع تعليمات الطبيب بشأن جرعتك وتوقيت تناول الدواء.<br>تذكر أنه من المهم الالتزام بالجرعات الموصوفة وعدم زيادة الجرعة دون استشارة الطبيب.<br>التفكير بالعرض الكبير:<br>احتضن فكرة أن هذه المناسبة ستمر بسرعة، وأنك ستكون قادرًا على التعامل معها بشكل جيد.<br>أتمنى أن تساعدك هذه الاقتراحات في التخفيف من القلق والتوتر قبل المناسبة غدًا. لا تتردد في اللجوء إلى أي من هذه الطرق التي تعمل بشكل جيد بالنسبة لك. في النهاية، حاول أن تكون لطيفًا مع نفسك وتقدم قدر الإمكان من الراحة والدعم لنفسك في هذه اللحظة.	Before the event, try deep breathing techniques and simple meditation to calm down and relieve anxiety.<br>Progressive muscle relaxation can also be helpful, where you focus on tensing and relaxing muscles sequentially.<br>Positive Thinking and Thought Guidance:<br>Try to replace negative thoughts with positive ones. Tell yourself that you can successfully handle these situations.<br>It might be useful to repeat positive affirmations to yourself, such as "I am strong and capable of overcoming this anxiety."<br>Talk to a Trusted Person:<br>If you can talk to a trusted person before the event, this might help relieve the pressure. Share your feelings and don't hesitate to ask for support.<br>Event-Oriented Techniques:<br>Before the event, try using event-oriented visualization techniques.<br>Imagine yourself successfully handling the awkward or stressful situation.<br>Leveraging Medication Support:<br>If you are using medication to control anxiety, make sure to follow your doctor's instructions regarding your dosage and timing of medication.<br>Remember that it's important to stick to the prescribed doses and not increase the dosage without consulting your doctor.<br>Focus on the Big Picture:<br>Embrace the idea that this event will pass quickly, and that you will be able to handle it well.<br>I hope these suggestions help you alleviate your anxiety and stress before tomorrow's event. Don't hesitate to resort to any of these methods that work well for you. Ultimately, try to be kind to yourself and provide yourself with as much comfort and support as possible in this moment.
(6) Being more or less informative	-	

(7) Ignoring expectations	المستخدم :هي دائما ما تشتكي بان هناك من لا يفهمها حتى لو أتكلم معها وأكون قد شاهدت فيديو يتكلم عن الشخصية الحدية وأتبع كل التعليمات معها وأفشل معها ولا تكون راضية<br>جي بي تي: إنه من المهم أن تعمل مع الاختصاصية النفسية على فهم تحديات ابنتك والأسباب التي تجعلها تشعر بأن هناك من لا يفهمها. قد يكون هذا جزءًا من التحديات التي تواجهها بسبب اضطراب الشخصية الحدية الذي تعاني منه. الاختصاصية يمكنها مساعدتك في فهم كيفية التعامل مع هذه الوضعية وتوجيهك بشكل مناسب حول كيفية دعم ابنتك.<br>قد يكون من الضروري تفهم أن العمل على تغيير السلوكيات وتطوير الشخصية يمكن أن يكون عملية طويلة وصعبة في بعض الأحيان. إذا كانت ابنتك تشعر بعدم الرضا حتى بعد *محاولاتها*، فهذا مؤشر على أنها قد تحتاج إلى دعم نفسي إضافي. الاختصاصية يمكنها تقديم نصائح وأساليب تساعد في تحسين فهمها للذات وتعزيز شعورها بالثقة.<br>تذكري دائمًا أن الدعم والتفهم من قبلك كوالدين لها أمران مهمان جدًا. قد تكون مهمتكما الرئيسية هي تقديم الدعم العاطفي والمحبة لابنتك، سواء تعاملت مع تغييرات في سلوكها أم لا. يجب أن تكونوا متواجدين لها ومستعدين للاستماع إليها وتقديم الدعم على مر الرحلة.	User: She always complains that no one understands her, even if I talk to her and I've watched videos about Borderline Personality Disorder and followed all the instructions, I still fail with her, and she's not satisfied.<br>GPT: It's important to work with a psychological specialist to understand your daughter's challenges and the reasons she feels misunderstood. This could be part of the difficulties she faces due to the Borderline Personality Disorder she suffers from. The specialist can help you understand how to deal with this situation and guide you appropriately on how to support your daughter.<br>It may be necessary to understand that working on changing behaviors and developing personality can sometimes be a long and difficult process. If your daughter feels dissatisfied even after her *attempts*, this is an indicator that she may need additional psychological support. The specialist can offer advice and methods that help improve her self-understanding and enhance her sense of confidence.<br>Always remember that your support and understanding as a parent are very important. Your main task may be to provide emotional support and love to your daughter, whether she deals with changes in her behavior or not. You should be present for her, ready to listen, and offer support throughout the journey. (The user never talks about her daughter's attempts to treat herself)
(8) Providing more information than what is required	-	-
(9) Self-contradiction	المستخدم: هل تنصحني بالتوقف عنها؟<br>جي بي تي :*كوني أنا موديل لغة اصطناعية تم تطويرها بواسطة أوبن إي آي، فإنني لا أستطيع تقديم النصائح الطبية الشخصية*. ومع ذلك، يمكنني تقديم بعض النصائح العامة.	User: Do you advise me to stop taking them?<br>GPT: *As an artificial language model developed by OpenAI, I cannot provide personalized medical advice.* However, I can offer some general advice. (The model gave some advice on Escitalopram in its earlier responses in the same consultation)

(10) Contradiction	المستخدم... :ولكن هولاء البنات ال 7 اللي هي تحارب من أجل التواصل معهم كملوا طريقهم ووصلوا *السنة رابعة طب* وصار عندهم أشغال ومشغولين...<br>جي بي تي: من الواضح أن ابنتك تمر بفترة صعبة وتجرب تحديات نفسية خطيرة. من الضروري التصرف بحذر وفورًا لدعمها ومساعدتها على التغلب على هذه المشكلة. إليك بعض الخطوات التي يمكنك اتخاذها:<br>التعاون مع *المدرسة* والأصدقاء تواصلي مع *المدرسة* وحاولي فهم ما يحدث هناك. قد يكون هناك معلومات تفيد في معرفة ما يجري.	User: …But those seven girls that she's fighting to connect with have continued their path, reached their fourth year of *medical school*, and are now working and busy…<br>GPT: It's clear that your daughter is going through a difficult period and experiencing serious psychological challenges. It's crucial to act cautiously and immediately to support her and help her overcome this problem. Here are some steps you can take:<br>Collaborate with the *school* and friends Communicate with the *school* and try to understand what's happening there. There might be information that helps clarify the situation. (The Arabic word for "school" is madrasah (المدرسة), and it strictly means education before college level. On the other hand, education above school level is referred to in Arabic using the words kulˈliːjah (كليّة) and d͡ʒaːmiʕah (جامعة). However, in English, school is appropriate in both contexts)
(11) Repetition	المستخدم :عندي مناسبة بعد كم يوم ومن وقت الدعوة الى الان وانا في حالة ما يعلم فيها الا الله حتى جهازي الهضمي انهار تماما من الخوف يعني معدة حموضة و قولون<br>جي بي تي :أعتذر للإحساس بالضغط والتوتر الشديد. إنه شعور طبيعي في مثل هذه الحالات، ولكن هناك بعض الطرق التي قد تساعد في التخفيف من هذه الأعراض قبل المناسبة:<br>*التنفس والاسترخاء* :حاول تنفيس الهواء ببطء وعميقًا عندما تشعر بالقلق. جلسة قصيرة من التأمل أو اليوغا يمكن أن تساعد في تهدئة الأعصاب.<br>المستخدم :عطوني طقوسكم قبل الذهاب لمثل هذه المناسبات و لا تبخلون علي باي نصيحة<br>جي بي تي :*التنفس والاسترخاء* :<br>قبل الذهاب، خذ وقتًا للتنفس بعمق وببطء. جرب تقنيات التأمل البسيطة لتهدئة أعصابك وتركيز عقلك.	User: I have an event in a few days, and ever since I received the invitation, I've been in a state that only God knows about. My digestive system has completely broken down from fear—I'm dealing with stomach issues, acidity, and IBS.<br>ChatGPT: I apologize for the intense pressure and stress you're feeling. It's a natural reaction in such situations, but there are some methods that might help alleviate these symptoms before the event:<br>*Breathing and Relaxation*<br>Try to exhale slowly and deeply when you feel anxious. A short session of meditation or yoga can help calm your nerves.<br>User: Give me your rituals before going to such events, and don't hold back any advice.<br>ChatGPT: *Breathing and Relaxation*<br>Before you go, take some time to breathe deeply and slowly. Try simple meditation techniques to calm your nerves and focus your mind.
(12) Ambiguous words and expressions	المستخدم :عندي مناسبة بعد كم يوم ومن وقت الدعوة الى الان وانا في حالة ما يعلم فيها الا الله حتى جهازي	User: I have an event in a few days, and ever since I received the invitation,

	الهضمي انهار تماما من الخوف يعني معدة حموضة و قولون<br>جي بي تي :التغذية الصحية: حاول تجنب الأطعمة *الثقيلة* والحارة والمشروبات المحتوية على الكافيين والكحول. تناول وجبات صغيرة ومتكررة قد تخفف من حدة مشاكل الهضم.	I’ve been in a state that only God knows about. My digestive system has completely broken down from fear—I’m dealing with stomach issues, acidity, and IBS.<br>ChatGPT: Healthy Nutrition. Try to avoid *heavy*, spicy foods and drinks containing caffeine and alcohol. Eating small, frequent meals might ease the severity of your digestive issues.
(13) Lack of sociality	المستخدم: أحاول قد ما اقدر أعمل رياضة أحيانا امشي في الأسبوع مرتين او مره واحيانا ولا مره هذا لما يكون الجو حار بس هذه الفترة الجو حلو امشي نص ساعة في الصباح اول ما يطلعوا اولادي اروح امشي<br>جي بي تي: من الرائع أنك تقوم بممارسة الرياضة بانتظام، حيث إن النشاط البدني يمكن أن يكون له تأثير إيجابي على المزاج والصحة العقلية بشكل عام. الاستمرار في ممارسة التمارين الرياضية بانتظام يمكن أن يساعد على تخفيف أعراض الاكتئاب.<br>إلى جانب ذلك، هناك بعض الخطوات الأخرى التي يمكنك اتخاذها للتعامل مع الاكتئاب:<br>1. الحفاظ على نمط حياة صحي: حاول الحصول على قسط كافٍ من النوم، وتناول وجبات صحية متوازنة، وتجنب الإفراط في تناول المشروبات المحتوية على الكافيين أو *الكحول*.	User: “I try my best to exercise. Sometimes I walk twice a week, or once, and sometimes not at all when the weather is hot. But the weather is nice now, so I walk for half an hour in the morning as soon as my kids leave.”<br>ChatGPT: “It’s wonderful that you’re exercising regularly, as physical activity can have a positive effect on your mood and overall mental health. Continuing to exercise consistently can help alleviate symptoms of depression. Besides that, here are some other steps you can take to deal with depression: Maintain a healthy lifestyle: Try to get enough sleep, eat healthy, balanced meals, and avoid excessive consumption of caffeinated beverages or *alcohol*.”
(14) Lack of common sense	المستخدم :أستخدم *موانع للحمل* هل ممكن تكون سببت الاكتئاب؟<br>جي بي تي: ومع ذلك، يجب أن نلاحظ أن العديد من العوامل يمكن أن تسهم في الاكتئاب، ولا يمكن تحديد العامل الفردي الذي يسبب الاكتئاب بالضبط. بالإضافة إلى ذلك، هناك أنواع مختلفة من وسائل منع الحمل، وقد تؤثر كل نوع بشكل مختلف على *الأفراد*.	User: “I’m using *contraceptives*. Could they be causing depression?”<br>GPT: “However, it’s important to note that many factors can contribute to depression, and it’s not possible to pinpoint a single cause. Additionally, there are different types of contraceptives, and each type may affect *individuals* differently.” (Only women can conceive)

Note. Communication errors are given in italics.
